# To PCR or not? The impact of shifting policy from PCR to rapid antigen tests to diagnose COVID-19 during the omicron epidemic: a nationwide surveillance study

**DOI:** 10.3389/fpubh.2023.1148637

**Published:** 2023-07-20

**Authors:** Hsin Chi, Nan-Chang Chiu, Chung-Chu Chen, Shun-Long Weng, Chi-Hone Lien, Chao-Hsu Lin, Yao-Feng Hu, Wei-Te Lei, Yu-Lin Tai, Liang-Yen Lin, Lawrence Yu-Min Liu, Chien-Yu Lin

**Affiliations:** ^1^Department of Pediatrics, MacKay Children's Hospital, Taipei City, Taiwan; ^2^Department of Medicine, MacKay Medical College, Taipei City, Taiwan; ^3^Department of Internal Medicine, Hsinchu MacKay Memorial Hospital, Hsinchu, Taiwan; ^4^Teaching Center of Natural Science, Minghsin University of Science and Technology, Hsinchu, Taiwan; ^5^Department of Obsterics and Gynecology, Hsinchu MacKay Memorial Hospital, Hsinchu, Taiwan; ^6^Department of Pediatrics, Hsinchu MacKay Memorial Hospital, Hsinchu, Taiwan; ^7^Department of Pediatrics, Hsinchu Municipal MacKay Children's Hospital, Hsinchu, Taiwan; ^8^Department of Biological Science and Technology, National Yang Ming Chiao Tung University, Hsinchu, Taiwan; ^9^Department of Laboratory, Hsinchu MacKay Memorial Hospital, Hsinchu, Taiwan; ^10^Pei Ying Junior High School, Hsinchu, Taiwan

**Keywords:** COVID-19, omicron, polymerase chain reaction, rapid antigen test, SARS-CoV-2, policy

## Abstract

**Background:**

Coronavirus disease 2019 (COVID-19) had caused huge impacts worldwide. Polymerase chain reaction (PCR) is the mainstay diagnostic modality. In most hospitals in Taiwan, samples for PCR are collected at emergency department (ER) or outdoor clinics to avoid virus spread inside hospitals. Home rapid antigen test (RAT) is a feasible, low-cost, and convenient tool with moderate sensitivity and high specificity, which can be performed at home to reduce hospital visits. Due to comparably low severity of omicron variant and high vaccine coverage (~80% residents fully vaccinated with AstraZeneca, Moderna, or Pfizer BioNTech COVID-19 vaccines as of March 2022), the policy was shifted from containment to co-existing with COVID-19 in Taiwan. Virus spread rapidly in the community after the ease of social restrictive measurements. To acquire a confirmed diagnosis, PCR testing was requested for people with suspected COVID-19 infection. As a consequence, people with respiratory symptoms or contact history surged into hospitals for PCR testing, thus, the medical capacity was challenged. The diagnostic policy was altered from PCR to RAT, but the impact of diagnostic policy change remains unclear.

**Objectives:**

We conducted this study to investigate the number of COVID-19 cases, PCR testing, hospitalizations, mortalities, and hospital visits during the epidemic and evaluate the impact of diagnostic policy change on hospital visits.

**Methods:**

The diagnostic policy change was implemented in late May 2022. We used nationwide and hospital-based data of COVID-19 cases, PCR testing, hospitalizations, mortalities, and hospital visits before and after policy change as of 31 Jul 2022.

**Results:**

During the omicron epidemic, significant and synchronous increase of COVID-19 patients, PCR testing, hospital visits were observed. COVID-19 cases increased exponentially since April 2022 and the COVID-19 patients peaked in June (1,943, 55,571, and 61,511 average daily new cases in April, May, and June, respectively). The PCR testing peaked in May (85,788 daily tests) with high positive rate (81%). The policy of RAT as confirmatory diagnosis was implemented on 26 May 2022 and a substantial decline of PCR testing numbers occurred (85,788 and 83,113 daily tests in May and June). People hospitalized for COVID-19 peaked in June (821.8 patients per day) and decreased in July (549.5 patients). The mortality cases also peaked in June (147 cases/day). This trend was also validated by the hospital-based data with a significant decrease of emergency department visits (11,397 visits in May while 8,126 visits in June) and PCR testing (21,314 in May and 6,158 in June). The proportion of people purely for PCR testing also decreased (10–26 vs. 5–14%, before and after policy change, respectively).

**Conclusions:**

The impact of diagnostic policy change was a complicated issue and our study demonstrated the huge impact of diagnostic policy on health seeking behavior. The PCR testing numbers and emergency department visits had substantial decrease after diagnostic policy change, and the plateau of epidemic peak eased gradually in ~1 month later. Widespread RAT application may contribute to the decreased hospital visits and preserve medical capacity. Our study provides some evidences for policy maker's reference.

## 1. Introduction

The long-running battle against the coronavirus disease 2019 (COVID-19) pandemic has entered the 3rd year, while human life has been substantially affected in all aspects (1, 2). The virus continues to evolve, and among the variants, the omicron variant is highly contagious and less severe (3, 4). Furthermore, vaccines against severe acute respiratory syndrome coronavirus 2 (SARS-CoV-2) have become widely available, and vaccinated individuals have a significantly lower risk of severe complications from COVID-19 (5, 6). Although the efficacy of the COVID-19 vaccines is less effective for omicron variant, vaccination remains effective to reduce severe complications after infection (7). Antiviral agents are beneficial for older adults, immunocompromised hosts, and other high-risk groups, meanwhile, timely diagnosis to allow early treatment is crucial to improve clinical outcomes (8, 9). Therefore, reconsidering the policy of a stringent control strategy to contain and control the pandemic is emerging in many countries (10). In 2022, the policy of coexisting with COVID-19 was adopted in Taiwan, and easing of containment strategies was implemented step by step (10, 11). The gold standard of COVID-19 diagnosis is polymerase chain reaction (PCR) which is widely adopted in Taiwan. To ensure the quality of PCR sampling, reduce nosocomial viral spread, and prevent transmission to susceptible and high risk patients inside hospitals, PCR sampling was performed at emergency departments (ER) or outdoor clinics in most Taiwan hospitals (12, 13). Walk-in clinics and drive-through testing stations are not widely available in Taiwan. This PCR-based strategy can decrease false-positive and negative rates of RAT, but people may surge into hospitals to receive PCR testing and thus cause collateral damage to people without COVID-19. The home rapid antigen test for SARS-CoV-2 (RAT) is a convenient tool with moderate sensitivity and high specificity when comparing with PCR (14, 15) and can be performed at home. To ensure early detection of infection cases and initiation of appropriate infectious control measures, RATs were not used in the initial phases of pandemic in Taiwan. During the process of reopening and coexistence, the number of COVID-19 cases increased exponentially, and medical capacity was challenged. The medical system may collapse during an epidemic surge (16–18). Therefore, the application of RATs to replace PCR as confirmatory tests was considered to decrease unnecessary hospital visits. In late May, the policy to diagnose COVID-19 changed from PCR testing to RATs. However, the impacts of shifting the diagnostic policy on health-seeking behaviors remain largely unclear.

By March 2022, ~80% of residents in Taiwan had been fully vaccinated (11). There has been an omicron epidemic in Taiwan since April 2022. There were 0.88 new cases daily per million residents on 01 Jan 2022 and 5,404.63 new cases on 14 May 2022 (2, 10). Although the majority of COVID-19 cases are mild and do not require hospitalization (4, 19), a rapid increase in cases is associated with a rapid increase numbers in hospitalizations and mortalities, especially for high-risk people (4, 20, 21). Furthermore, PCR testing played important roles in many scenarios at that time, including diagnosis confirmation, prescription of antiviral agents, admission routine tests, and proof for insurance payments. Confirmation of COVID-19 by PCR testing was required at that time to establish the diagnosis and prescribe antiviral medication in a timely manner. All hospitalized patients were requested to receive PCR testing before admission to reduce nosocomial transmission of SARS-CoV-2. Moreover, proof of PCR testing was necessary for quarantine, pandemic leave, and insurance payments. People with confirmed infection had to be quarantined for 7 days to reduce disease spread in the community and a proof by PCR testing was needed for schools and companies. Additionally, several companies unveiled an insurance policy with an NTD$500 (~17 USD) payment and NTD$50,000 payout if the individual had to quarantine. Therefore, during the omicron epidemic, people with fever, respiratory symptoms, or a history of contact with COVID-19 patients surged into the emergency department to asked for PCR testing. As a result, the rapid increase in the number of patients became a big challenge for the medical system, and thus resulted in the impending collapse of medical services, especially in emergency departments. Shifting diagnostic policy from PCR to RATs may decrease the need for emergency visits and preserve medical capacity. We conducted this retrospective study using nationwide and hospital data of COVID-19 patients, including diagnostic testing, hospitalizations, mortalities, and hospital visits. We investigated the epidemiological trends before and after the policy change to evaluate the impacts of the diagnostic policy change from PCR testing to RATs.

## 2. Materials and methods

### 2.1. Study design and data collection

COVID-19 is a communicable disease, and all confirmed cases will be reported to Taiwan Centers for Disease Control and Prevention per the domestic regulation. We retrospectively collected epidemiological data from public data sources and our hospital-based visits (2, 10). First, we extracted epidemiological information regarding the daily new COVID-19 cases, the COVID-19 vaccination rate, daily new tests, the PCR positive rate, and the mortality cases from the open access website OurWorldInData.org (2). The definition of new case evolved by time. New cases were diagnosed by PCR before late May 2022 and then diagnosed by both PCR and RAT afterwards. The PCR results were reported to the government by medical care units; after diagnostic policy change, the positive RAT results could be reported to the government via telemedicine or by medical care units. The mortality cases referred to deaths of patients with positive tests without obvious alternate causes. Fully vaccinated people refer to people with two doses of COVID-19 vaccines or one dose of Johnson & Johnson/Janssen vaccine. In Taiwan, there were several kinds of COVID-19 vaccines available in different periods, including AZD1222 (by AstraZeneca/Oxford, UK), mRNA-1273 (by Moderna, USA), BNT162b2 (by Pfizer/BioNTech, Germany), Nuvaxovid (by Novavax, USA), and MVC-COV1901 (by Medigen, Taiwan). COVID-19 vaccines were freely provided to residents and full vaccination indicated two doses of homologous or heterologous administration of above vaccines. A booster referred to a third dose of mRNA vaccine (mRNA-1273 or BNT162b2) or protein-based vaccine (Nuvaxovid or MVC-COV1901). The national hospitalizations were extracted from the website of Taiwan Centers for Disease Control (10). The 7-day average was summarized to decrease the artificial peaks or valleys observed on weekends. Second, we extracted the number of emergency department visits to our hospital since 2019 to validate the nationwide trend and investigated the impact of the omicron epidemic on patient visits. Finally, we compared the trend in PCR tests and emergency visits before and after the diagnostic policy change to evaluate the impacts of the policy change.

### 2.2. Ethical considerations

The present study was approved by the Institutional Review Board of the MacKay Memorial Hospital, Taipei, Taiwan (approval number 20MMHIS140e). We utilized database analysis, and no personal identifiable information was used in this study.

### 2.3. Statistical analyses

We plotted the trends for new COVID-19 cases, PCR tests, and patient visits using Microsoft Office, version 2019 (Microsoft Corp, New Mexico, USA). Linear regression analyses were performed using the equation of linear trend estimation. The slope of the regression line indicated a positive or negative change in trends. *R*^2^-values were also calculated, and a higher *R*^2^-value indicated lower discrepancies between datasets. Furthermore, we used independent *t*-tests to compare the monthly patient visits in the pre-epidemic and epidemic periods and the number of PCR tests before and after the policy change. An interrupted time series analysis was performed to evaluate the impact of the policy change intervention (22). A *p*-value < 0.05 indicated statistical significance. SPSS version 23.0 (IBM Corp, Armonk, NY, USA) and R software version 4.2.1 (R Foundation for Statistical Computing, Vienna, Austria) were used for statistical analyses.

## 3. Results

### 3.1. Epidemiology of COVID-19 patients and ER visits

**Figure 1:**
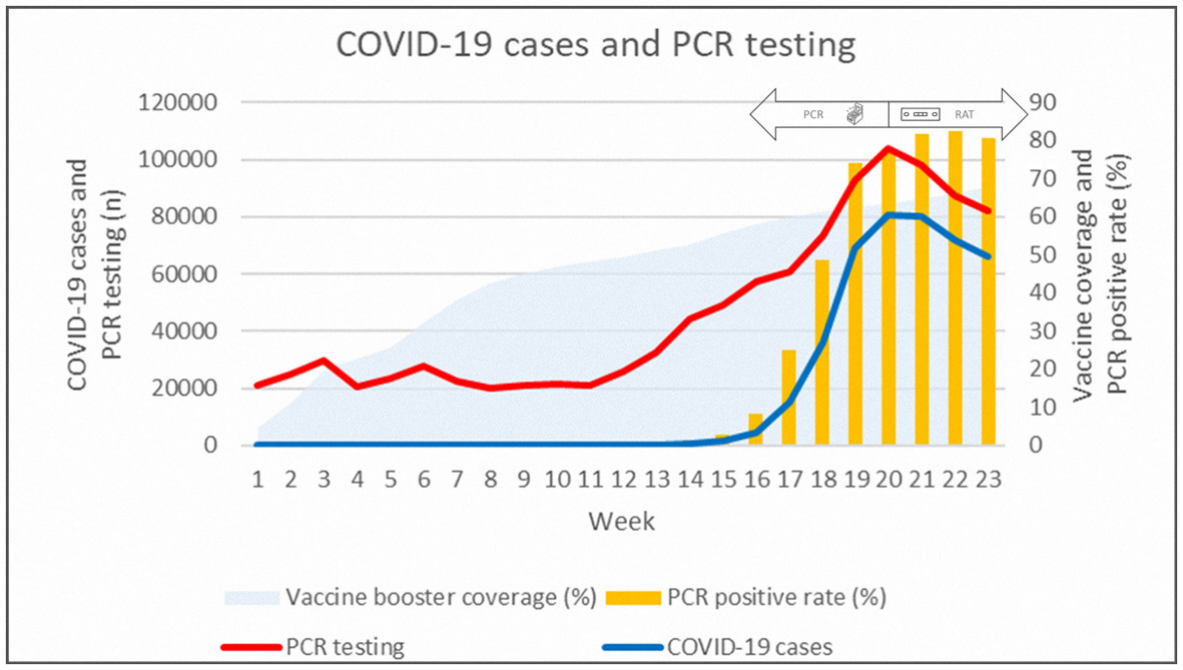
Epidemiological data of daily new COVID-19 cases, PCR testing, PCR positive rate, and vaccination booster coverage rate in 2022.

Figure 1 summarizes the epidemiological trend in 2022. As of 31 July 2022, there were 458,185 confirmed cases of COVID-19 in Taiwan (192,297 cases per million residents), and ~80% of residents were fully vaccinated, with 22% boosted. The booster coverage increased to ~65% by week 23. Both the confirmed new cases and the number of PCR tests increased exponentially after week 16 (April) and peaked by week 20. The number of daily PCR tests decreased after the policy change, and the peak of new cases persisted for ~1 month. The positive rate of PCR testing increased by more than 80% after week 20.

The numbers of COVID-19 cases, PCR tests, hospitalizations, mortalities, hospital ER visits, and hospital PCR tests are summarized in Table 1. The surge in omicron infection cases began in April and peaked in June (1,943, 55,571, and 61,511 daily new cases in April, May, and June, respectively, Table 1). The plateau of the epidemic declined ~1 month later (28,101 daily new cases in July). The total number of national PCR tests also increased, with a peak in late May (85,788 daily tests). The number of COVID-19 hospitalizations and mortalities also peaked in June (822 hospitalizations daily for COVID-19 and 147 mortalities daily). The number of ER visits to our hospital fluctuated, with a small peak from October 2020 to March 2021 and October 2020. A significant increase was observed in May 2022. Compared with the same month, a more than half increase in monthly visits was observed in May 2022 (2020: 5,333, 2021: 6,471, 2022: 11,397 visits). ER visits decreased in June 2022 (11,397 visits). The proportion of visits purely for PCR testing also decreased (26, 10, and 5% in April, May, and June, respectively).

**Table 1:** Average daily numbers of national COVID-19 patients, PCR testing, hospitalizations, mortalities, monthly hospital PCR testing, outpatient clinic visits, and emergency department visits.

	**Year**	**Jan**	**Feb**	**Mar**	**Apr**	**May**	**Jun**	**Jul**	**Aug**	**Sep**	**Oct**	**Nov**	**Dec**
**COVID-19 patients (cases/day)**													
	2020	1.04	0.88	8	4.95	0.41	0.18	0.54	0.78	0.8	1.3	2.94	4.85
	2021	3.47	1.81	2.32	3.01	195.04	248.47	32.56	11.12	7.71	6.3	6.06	12.17
	2022	53.7	60.06	83.61	1,942.67	55,570.67	61,510.75	28,100.91	22,622.18				
**National PCR testing (tests/day)**													
	2020	66	301	669	1052	326	154	154	188	208	262	272	501
	2021	862	787	465	579	8,711	27,466	22,924	20,767	23,198	19,241	16,326	15,765
	2022	23,111	22,939	21,747	45,884	85,788	83,113^*^						
**National COVID-19 hospitalizations (7-day average**, ***n*****/day)**													
	2022	287.4	221.3	200.1	436.7	747.1	821.8	549.5					
**National COVID-19 mortality cases (** ***n*** **/day)**													
	2022	0.03	0.06	0.01	0.2	34.1	147.1	79.1	33.9				
**Hospital monthly PCR testing (tests/month)**													
	2020			993	1,772	299	128	209	412	300	654	558	570
	2021	963	1,684	1,062	946	2,695	5,672	5,648	4,899	5,035	5,257	4,494	3,506
	2022	7,340	6,558	7,840	13,209	21,314	6,158	5,468	5,958				
**Hospital monthly OPD visits (patient visits/month)**													
	2020	54,103	53,424	53,553	49,848	58,826	62,899	67,391	64,548	66,669	67,396	65,310	68,340
	2021	59,951	46,717	69,450	65,368	52,972	43,804	57,423	60,541	60,957	65,100	66,145	66,666
	2022	64,159	50,490	76,842	60,765	55,049	58,063	62,799	67,254				
**Hospital monthly ER visits (patient visits/month)**													
	2020	8,774	5,694	5,043	4,987	5,333	5,795	6,071	6,552	6,431	8,042	7,813	6,982
	2021	7,049	6,923	7,152	6,728	6,471	5,715	5,720	6,167	6,602	7,343	6,847	6,034
	2022	7,480	6,744	7,778	7,520	11,397	8,126	7,503	7,945				
2022 purely for PCR (%)		1,626 (22%)	1,679 (25%)	1,570 (20%)	1,985 (26%)	1,114 (10%)	406 (5%)	1,023 (14%)	1,141 (14%)				

* As of 22 Jun 2022.

COVID-19, coronavirus disease 2019; ER, emergency department; OPD, outpatient clinic department; PCR, polymerase chain reaction.

**Figure 2:**
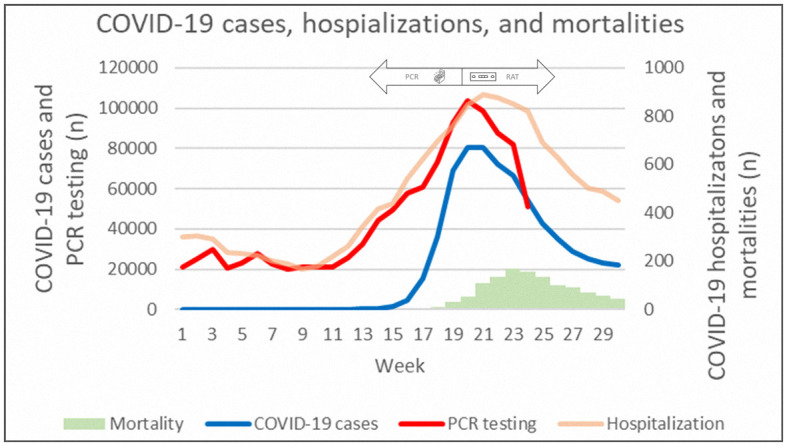
Epidemiological data of daily new COVID-19 cases, PCR testing, hospitalizations, and mortalities.

Figure 2 demonstrates the epidemiological trends in COVID-19 cases, PCR testing, hospitalizations, and mortalities. The peak of PCR tests was week 20, and the peaks of daily new cases, hospitalization, and mortalities followed.

**Figure 3:**
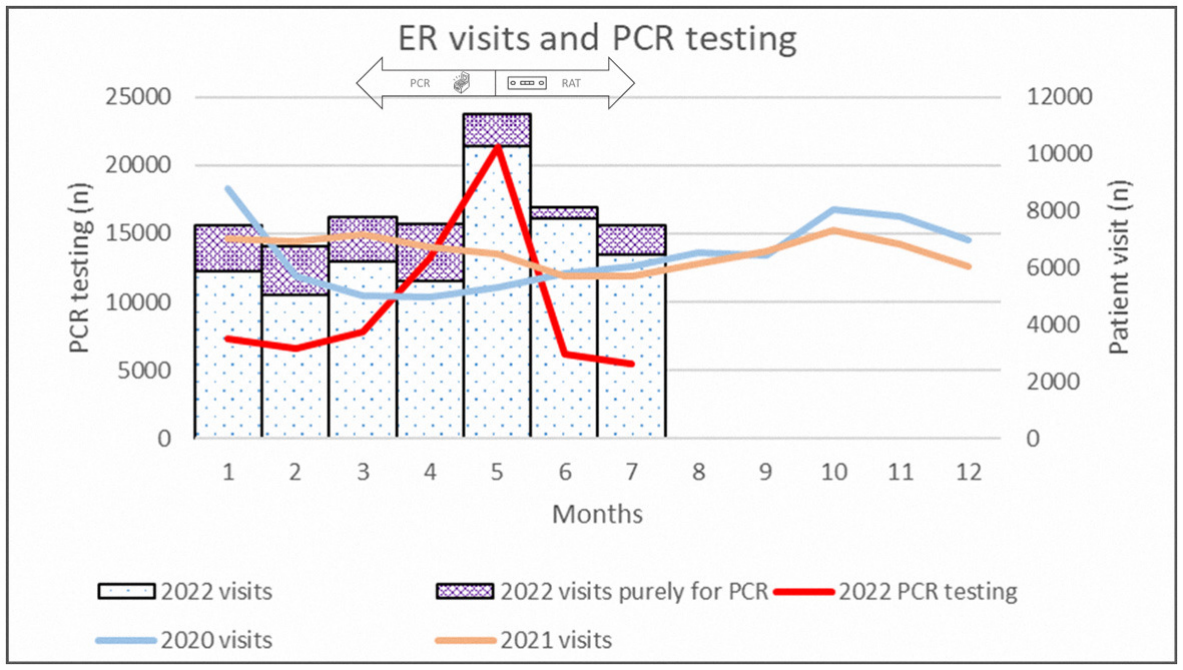
Epidemiological data of emergency department visits between 2020 and 2022 and PCR testing in 2022.

Figure 3 shows the monthly ER visits at our hospital. After the beginning of the pandemic in 2020, ER visits declined substantially. A small peak was observed after the end of the pandemic between October 2020 and March 2021. The number of ER visits increased rapidly after April 2022 and declined after the policy change in late May. The proportion of ER visits purely for PCR testing also declined after the policy change.

### 3.2. Impact of the policy change on testing

**Figure 4:**
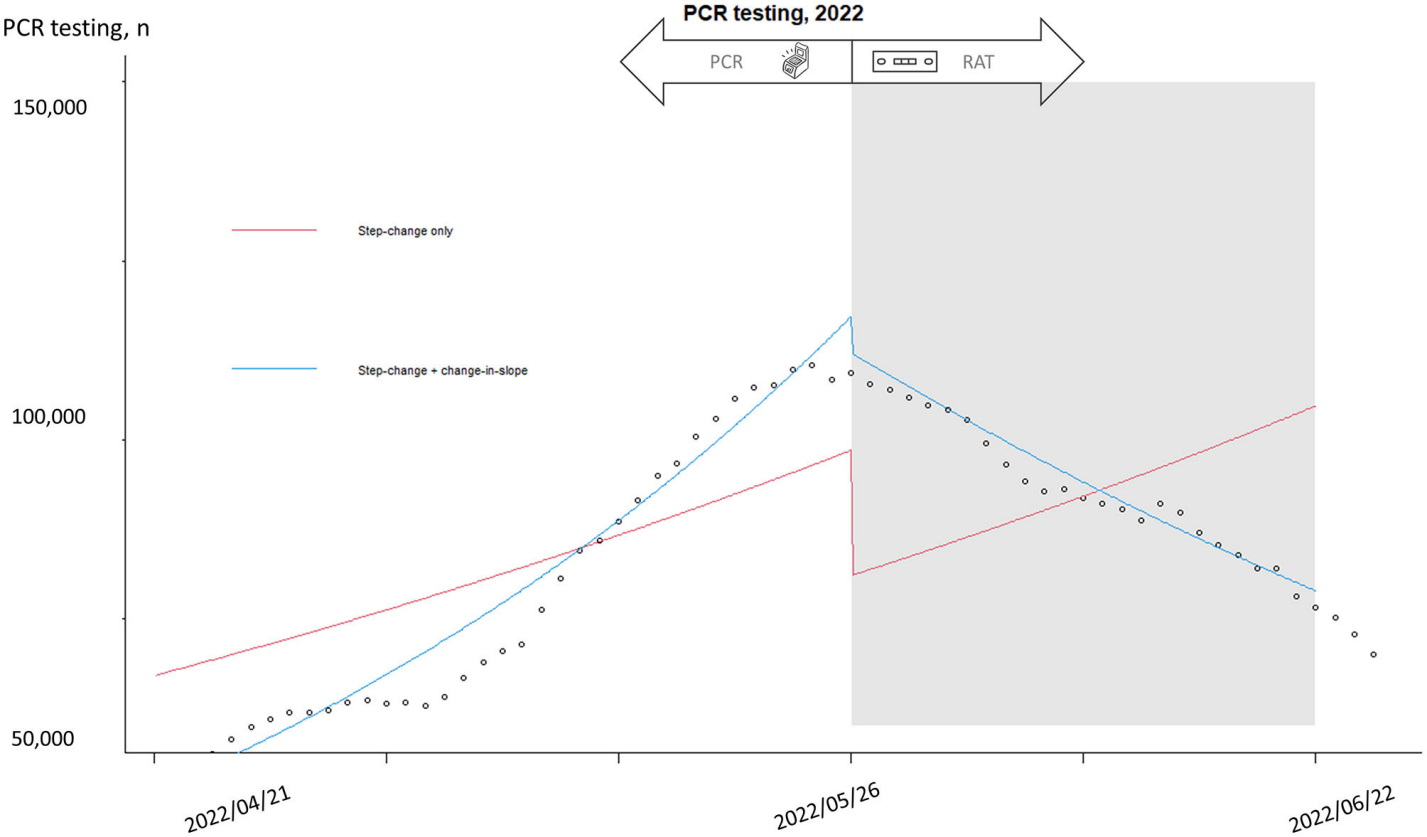
Interrupted time series analysis of national PCR testing before and after policy change. Interrupted time series was used to predict the trend with and without intervention. “Step-change only” (red line) refers to the impact of intervention made difference at one time point and a significant reduction of PCR testing occurred after policy change. “Step-change and slope change” (blue line) refers to a continuous impact after intervention and a steady decrease of PCR testing was observed after policy change.

Table 2 summarizes the trends in new COVID-19 patients, nationwide PCR testing, hospitalizations, mortalities, and hospital-based PCR testing. Negative trends were observed after the implementation of diagnostic policy changes, especially for new COVID-19 cases and hospitalizations. Furthermore, interrupted time series analysis showed a significant difference after the intervention (Figure 4). The black circles indicate the number of national daily PCR tests, with the peak in May. The blue lines demonstrate the independent trends before and after implementation of the policy change, and a significant reverse trend was observed. The red line indicates the same trend (slope) throughout the whole study period, and the step change indicates the impact of the policy change as a single episode. A substantial reduction in PCR testing was observed as a step change after implementation of the policy change.

**Table 2:** Linear trend in daily national COVID-19 cases, PCR testing and hospital PCR testing before and after the policy change.

	**Before policy change (01 Apr 2022–26 May 2022)**			**After policy change (27 May 2022–31 July 2022)**		
	**Average**	**Slope**	***R* ^2^**	**Average**	**Slope**	***R* ^2^**
National COVID-19 cases (daily *n*/million)	24,528	69.8	0.8	47,338	−44.8	0.96
National PCR testing (daily *n*/thousand)^*^	2,640	0.06	0.95	3,623	−0.05	0.97
National COVID-19 mortalities (*n*)	10.66	0.848	0.64	111.1	−1.369	0.47
National COVID-19 hospitalizations (*n*)	580.2	10.43	0.98	699.7	−8.213	0.94
PCR positive rates (%)^*^	28.4%	0.018	0.86	81%	−0.0013	0.39
Hospital PCR testing (*n*)	587.2	6.88	0.04	201	−1.64	0.17

COVID-19, coronavirus disease 2019; PCR, polymerase chain reaction. The * indicated the data of PCR testing after policy change was calculated as of 22 Jun 2022.

## 4. Discussion

Our study demonstrated exponential increases in the numbers of COVID-19 cases, hospitalizations, and mortalities during the omicron epidemic. PCR testing was the mainstay diagnostic modality and sharp increase of PCR testing coincided with the increase in COVID-19 cases. The number of PCR tests peaked in May, and the numbers of COVID-19 cases, hospitalizations, and mortalities peaked in June. The diagnostic policy change from PCR to RATs may have contributed to the reduction in PCR testing and unnecessary hospital visits and preservation of medical capacity. Our findings provide evidence that can be used as a reference for policy-makers.

COVID-19 continues to be an important threat in many countries, and the complicated multidirectional interactions between COVID-19 infection, the medical system, health policy, and human behaviors remain largely unclear (19, 23). Although many people avoided unnecessary hospital visits to decrease the risk of infection (for example, a significant reduction in childhood vaccinations was reported in many countries) (24), the rapid increase in COVID-19 patients may have resulted in the collapse of public health and medical systems (16, 17, 20, 21). Lockdown strategies, a shortage of human resources for illness or quarantine, and a lack of medication and facilities will aggravate the disruption of medical services (25). Although the prevalence of many respiratory infection decreased during the pandemic, we observed small peaks from October 2020 to March 2021 and October 2020. Outbreaks of respiratory syncytial virus may contributed to the observed peaks (26). During the omicron epidemic, our study showed synchronous increases in COVID-19 cases, hospitalizations, and mortalities, and we also found a significant increase in ER visits. After the diagnostic policy was changed from PCR testing to RATs, a significant decrease in ER visits and PCR testing was observed, emphasizing the importance of diagnostic policy in health-seeking behaviors.

During the initial waves of COVID-19, Taiwan adopted “containment” strategies to reduce virus spread and disease burden, and several aggressive and stringent strategies were employed, including border control, proactive testing, and quarantine (10, 12, 13, 27, 28). Based on the increased transmissibility and lower severity of the omicron variant, many countries adopted a “coexisting” policy in 2022, including Taiwan. The omicron variant is highly contagious, and the number of COVID-19 cases increased exponentially (4, 29). Infected people surged into hospitals, and the capacity of medical services was challenged. Fortunately, most people with omicron infection were less severely ill and did not need hospitalization. High coverage of COVID-19 vaccination also reduced the impact of patient surge after ease of social restrictive measurements (30). However, crowds of patients may lead to the collapse of medical services, especially in emergency departments. People had to wait for more than 2 h to undergo PCR testing, and it was difficult to maintain safe distancing in the ER. The risk of catching the infection at the ER and the ostracization of other medical needs should be considered. The road to peaceful coexistence in the omicron era could be painful, and efforts have been made to reduce collateral injuries (4). During the epidemic waves, coincident increases in cases and hospitalizations were reported in previous studies, and mortalities peaked in ~1 month (4, 6, 19, 21, 31). Hospitalizations and mortalities were complicated and might reach a peak after 1 month of infection peak. Our study showed similar trends, and all new cases, hospitalizations, and mortalities reached their peak in June. Diagnostic policy change didn't change the epidemiological trends, including peaks of new case, hospitalizations, and mortalities. Studies comparing PCR testing are scarce, and it is intuitive that the consumption of PCR tests coincided with the epidemic wave. We found a significant reduction in PCR testing after the policy change, and the epidemic waves declined after 1 month. Although the observed interval between PCR testing and epidemic wave peaks were not long that the clear relationship between diagnostic policy change and health seeking behaviors was not easily identified. Early implementation of policy change may have a more significant and clear impact on the epidemic. Political, environmental, economic, and medical factors may affect medical-seeking behaviors, and our study demonstrated the potentially important role of diagnostic policy during the epidemic. Policy-makers should incorporate public responses into their decision-making process.

Medical insurance has played important roles in the COVID-19 pandemic, but the government and private insurance systems vary in different countries (32–34). During the COVID-19 epidemic, people have typically avoided unnecessary hospital visits to decrease the risk of infection. COVID-19 has had huge impacts on medical services and immunization to different degrees (24). However, medical-seeking behaviors are complicated and affected by many psychological, economic, environmental, and social factors. For example, medical service and vaccination interruptions were reported in many areas during the pandemic and both government-funded and self-paid vaccinations decreased in Taiwan. However, there was an delta epidemic in Taiwan in 2021 and there was inadequate COVID-19 vaccine supply. Under the circumferences, people believed the potentially collateral benefits of pneumococcal vaccination that pneumococcal vaccination contributed to prevent COVID-19 infection and subsequent pneumonia. As a consequence, a reverse increase in self-paid pneumococcal vaccinations was observed (24). During the omicron epidemic, an increase in ER visits was expected due to rapid spread of the virus, but hospital visits may also be affected by non-medical factors. Most patients had mild illness, and ER visits were not needed. Some patients with mild illness visited the ER for PCR testing, and our study showed a significant decline in ER visits after the policy change. COVID-19 is a communicable disease, and a confirmative diagnosis by PCR was required before 26 May 2022. PCR testing was essential for quarantine, office leave, school leave, and insurance payments. Therefore, people with contact history, fever, or respiratory symptoms surged into the ER for PCR testing. Moreover, socioeconomic disparities and a lack of health insurance are important public health issues in COVID-19 (35–37). People with a lower socioeconomic status or no health insurance might have poorer outcomes after infection (38). However, the impacts of COVID-19 on insurers may be conflicting. Life insurers had higher liability during the pandemic and negative impacts on life insurers' financial sustainability may occur for higher mortality rates than expected (39). On the other hand, unexpected health insurance profits were noted due to sharp declines in elective care (40). The special pandemic insurance in Taiwan unveiled an insurance policy with an NTD$500 (~17 USD) payment and NTD$50,000 payout if the individual had to quarantine. This policy earned huge profits for insurance companies in 2021 due to a low prevalence of COVID-19 infection. During the omicron epidemic in 2022, many people were infected, and the insurance company lost much money. The collateral injury of increased ER visits caught our attention, and our study showed a substantial impact of policy changes on PCR testing and ER visits. Policy-makers may take the potential effects into consideration.

Virus culture is time-consuming, and PCR testing is the gold standard for many viruses. Compared with PCR, the RAT is quick, feasible, cheap, and convenient, with moderate sensitivity and high specificity (14, 15, 41–43). For symptomatic patients, the sensitivity of the RAT is ~80%, with a high specificity of 98.9% (43). The estimated positive predicted value (PPV) is 94.1%, and the negative predicted value (NPV) is 95.9%. For asymptomatic people, the sensitivity decreases to 41.2%, and the specificity remains high (98.4%), with a lower PPV of 33.3% and a higher NPV of 98.8%. The performance of the RAT is also affected by sampling methods, kit brands, and diagnostic methods, among other factors (42). In early stages, stringent containment was used and Taiwan government didn't adopt RAT as confirmatory tests for the issues of false positivity and relatively compromised sensitivity. People surged into hospitals and had to wait for hours for PCR testing to be performed. Medical capacity was challenged, and medical collapse may occur during the omicron epidemic. Furthermore, the background prevalence also interferes with the diagnostic accuracy (44). During the overwhelming epidemic stage, the sensitivity of the RAT increased, and using the RAT as a first-line diagnostic tool became a reasonable strategy. We found a high PCR positive rate of more than 80% after week 20, and the need for PCR testing was questioned. This policy can decrease the consumption of PCR testing and provide a timely diagnosis and further quarantine. Physicians can still perform PCR testing for selected cases, such as patients with severe infection. Furthermore, rapid walk-in clinics or drive-through testing stations were adopted in some countries with good efficacy (45). According to our hospital-based data, up to 26% of ER visits were purely for PCR testing. The application of rapid drive-through testing services may provide further benefits for these individuals. Our study provided evidence that diagnostic policy changes can reduce PCR testing and hospital visits. Thus, medical capacity can be preserved for patients with moderate or severe infection.

The strength of our study is that it is the first study to investigate the impact of diagnostic policy change on PCR testing and ER visits in Taiwan. However, our study was subject to some limitations. First, many factors affect health-seeking behaviors, including perceived risks, feasibility of medical resources, family support resources, vaccination status, and feasibility and supply of RATs. We observed a peak gap between PCR testing and COVID-19 cases, but earlier implementation of policy change may present a more significant impact. The degree of the policy change impact is not easily justified. Second, the causal relationship and underlying mechanisms were not clarified. We observed an association between the decrease in PCR testing and ER visits and the policy change, but the roles of the insurance system and socioeconomic factors were not fully elucidated. We are unable to draw a direct conclusion. Finally, our study was conducted in a local hospital. Although national epidemiological data were used, nationwide surveillance data on patient visits would be valuable.

## 5. Conclusions

In conclusion, the omicron epidemic caused an exponential increase in cases and challenged the medical system in Taiwan in April and May 2022. We observed a significant reduction in PCR testing and ER visits after the diagnostic policy change from PCR testing to RATs. Declines in COVID-19 cases, hospitalizations, and mortalities occurred within ~1 month. The policy change may have had a huge impact on health-seeking behaviors and medical resources. Our study provides evidence that can be used as a reference for policy-makers.

## Data availability statement

The raw data supporting the conclusions of this article will be made available by the authors, without undue reservation.

## Author contributions

HC, N-CC, and C-YL: conceptualization. HC, C-CC, W-TL, and C-YL: formal analysis. HC, N-CC, C-CC, and S-LW: investigation. C-HsL and LY-ML: methodology. C-YL: writing-original draft. All authors: data curation, validation, contributed to and reviewed the final submitted manuscript, had full access to all the data in the study, and had final responsibility for the decision to submit for publication.
